# Comorbidities in Age-Related Cataract: Epidemiological Burden and Public Health Implications

**DOI:** 10.3390/vision10020024

**Published:** 2026-04-28

**Authors:** Matteo Ripa, Matteo Forlini, Chiara Schipa, Neeraj Apoorva Shah

**Affiliations:** 1 Department of Ophthalmology, Sankara Eye Hospital, Jaipur 302039, India; 2 Sankara Academy of Vision, Bangalore 560037, India; 3 Ophthalmology Department, San Marino State Hospital, 47893 Cailungo, San Marino; 4 Department of Emergency, Anesthesiological and Reanimation Sciences, Fondazione Policlinico Universitario A. Gemelli IRCCS, 00168 Rome, Italy; 5 Catholic University “Sacro Cuore”, 00168 Rome, Italy

**Keywords:** cataract, cataract surgery, epidemiology

## Abstract

Cataracts represent the leading cause of blindness worldwide, particularly in older adults, and constitute a significant public health challenge. Although cataract surgery is generally associated with a high safety profile, both patients and healthcare providers often face significant challenges due to age-related physiological changes and the high prevalence of comorbidities, which are directly linked to cataractogenesis and other systemic diseases that can complicate both the surgical procedure and postoperative recovery. This narrative review aimed to assess the epidemiological characteristics of age-related physiological and pathological comorbidities in older adults with cataracts, evaluating their impact on preoperative assessment, surgical outcomes, and public health planning. Articles were identified through non-systematic searches of PubMed, EMBASE, and Scopus using a combination of medical subject headings (MeSH) terms and free-text keywords. Among the multiple non-ocular comorbidities, carotid artery disease (CAD) and hypertension (HTN) are among the cardiovascular diseases (CVDs) with the highest correlations with cataract. Diabetes, dyslipidemia, and metabolic syndrome are also highly prevalent and significantly influence surgical outcomes, as poor glycemic control increases intraoperative risks and postoperative complications. Additionally, neurological conditions such as stroke, Parkinson’s disease, and epilepsy often complicate anesthesia administration, contribute to postoperative delirium, and affect adherence to treatment protocols. Given these complexities, a multidisciplinary approach and targeted preoperative screening may offer personalized care to improve safety and outcomes. Despite advances in clinical care, disparities in access to cataract surgery, especially in underserved populations, continue to exist. Thus, a coordinated public health strategy that promotes early detection, equitable access, and the integration of innovations such as teleophthalmology and artificial intelligence is essential to optimize care for older adults with cataracts worldwide.

## 1. Introduction

Around 20 million individuals globally suffer from cataracts, which represent over 51% of all blindness cases globally [1]. Cataract prevalence rises significantly with age; estimates suggest that over 60% of individuals will develop cataracts by age 70, and 80% by age 80 [2]. Specifically, several studies, including the Baltimore Eye Survey and the Beaver Dam Eye Study, reported a correlation between cataract prevalence and aging, showing that cataract incidence increases with each advancing decade of life [3,4]. As a result, the growing global older adult population and rising cataract cases pose a serious public health challenge [5]. Currently, cataract surgery is among the most commonly performed surgical procedures globally [6], and it can improve visual acuity, enhance overall quality of life, and reduce falls among older adults [7].

Despite cataract surgery being one of the most frequent surgical procedures, patients and healthcare providers frequently face numerous challenges due to age-related physiological alterations and the elevated rate of comorbidities among older adults. Comorbidities such as cardiovascular diseases (CVDs), diabetes, frailty, hypertension (HTN), and cognitive impairment significantly raise perioperative and intraoperative risk. Therefore, a proper preoperative assessment is essential for improved patient outcomes and requires a multidisciplinary evaluation to provide an integrated assessment of the patient’s condition [8,9,10,11,12,13,14,15,16,17,18,19,20,21,22,23,24,25,26,27,28,29,30,31,32,33,34,35,36].

To the best of our knowledge, no review focusing specifically on age-related cataracts has combined an epidemiological analysis of systemic comorbidities with a public health perspective. Therefore, our review aims to assess the epidemiological characteristics of systemic comorbidities in older adult patients with age-related cataracts, with particular attention to those associated with cataract development, those commonly observed in patients undergoing cataract surgery, and those that have a meaningful impact on perioperative risk and surgical outcomes. Additionally, we aimed to provide public health implications and recommendations to optimize care and management in this population.

## 2. Epidemiology of Non-Ocular Comorbidities

Older adults with cataracts often exhibit a variety of comorbidities affecting multiple organs and systems [8,9,10,11,12,13,14,15,16,17,18,19,20,21,22,23,24,25,26,27,28,29,30,31,32,33,34,35,36]. Many of these comorbidities, such as HTN, are directly associated with both cataractogenesis and other systemic diseases; consequently, they may complicate the surgical procedure and postoperative recovery [37]. However, not all comorbidities serve identical roles within the context of cataracts. Some are primarily implicated in disease pathogenesis; others denote the overall burden of illness in older adults undergoing surgery; and only a subset exerts a direct influence on perioperative risk and surgical outcomes. Importantly, many of these comorbidities reflect shared age-related processes and multimorbidity rather than a direct causal relationship with cataracts themselves and should therefore be interpreted within the broader context of aging.

### 2.1. Materials and Methods

To examine the epidemiology of systemic comorbidities in older adults with age-related cataracts and their impact on preoperative assessment, surgical outcomes, and public health planning, we conducted a narrative literature review based on a structured search strategy. Two authors (M.R. and C.S.) screened and collected, with the help of a third author (N.A.S.) in case of no consensus, articles that had been identified through non-systematic searches of PubMed, EMBASE, and Scopus databases using a combination of Medical Subject Headings (MeSH) terms and free-text keywords such as “age-related cataract,” “comorbidities,” “older adults,” “preoperative assessment,” and “public health.” Additionally, further relevant studies were identified through a manual screening of the reference lists of selected articles to ensure the inclusion of key papers that may not have been identified in the initial database search. When full-text articles were not accessible, we contacted the corresponding authors to request copies of eligible studies. No filters were initially applied to publication type or date to ensure a comprehensive overview. Original research articles, observational studies, population-based surveys, and clinical trials providing relevant quantitative or qualitative data and addressing the epidemiology of systemic comorbidities in older adults with cataracts, their implications for perioperative assessment or surgical outcomes, or broader public health strategies related to cataract surgery in aging populations were included. We excluded literature reviews, theses, book chapters, and conference abstracts from our analysis. The literature search was conducted up to March 2025. No restrictions on publication date were applied in order to provide a comprehensive overview of the available evidence. Although a structured search strategy was employed, this review was conducted as a narrative review and did not follow a formal systematic review methodology (e.g., PRISMA guidelines). Therefore, no formal study selection flow diagram or quantitative synthesis was performed. Studies were selected based on their contribution to the review’s conceptual framework.

### 2.2. Cardiovascular Comorbidities

The preoperative evaluation of older adults undergoing cataract surgery significantly relies on CVD evaluation because of the high prevalence of CVDs and their possible effects on surgical safety and outcomes [38]. According to currently available research, oxidative damage from free radicals, inflammation, and the accumulation of glycation products are the primary mechanisms through which cataractogenesis is connected to CVDs [8,9,37,39]. In 2010, Namet et al. [8] reported that several CVDs and cardiovascular risk factors were more common among patients undergoing cataract surgery. In addition, among CVDs, carotid artery disease (CAD) was found to have the highest correlation with cataracts. Indeed, since the carotid artery supplies blood to the eye, CAD can cause cataract formation by damaging ocular circulation. Moreover, numerous risk factors associated with CVDs not only impact the development of cataracts but also exacerbate pre-existing conditions. Consequently, these factors present significant challenges for older adults seeking cataract surgery, affecting both the surgical procedure and the subsequent recovery [8,9,37,39]. In 1999, Goodrich et al. studied the connection between cataracts and established cardiovascular risk factors, reporting that high plasma fibrinogen was an independent risk factor for cataracts because elevated fibrinogen levels could impair blood flow by promoting atheroma formation, platelet aggregation, and increased plasma viscosity [40]. Recently, emerging evidence has reinforced this concept, suggesting that hemostatic dysregulation and chronic low-grade inflammation play a key role in cataractogenesis through mechanisms involving microvascular impairment, oxidative stress, and altered ocular perfusion [41].

HTN represents a common comorbidity affecting about two-thirds of older adults with age-related cataracts [10]. Due to its implications in potential blood pressure (BP) fluctuations, it may be directly related to a high risk of intraoperative bleeding, retinal vein occlusion, or acute cardiovascular complications such as myocardial infarction or stroke [42,43]. The prevalence of HTN in older adults and its correlation with cataractogenesis have been extensively evaluated [11,12,44,45,46,47,48,49,50,51,52]. Recently, Prashant et al. discovered that HTN and diabetes accounted for a significant portion of comorbid patients, totaling around 78% of older adults with age-related cataracts. Furthermore, HTN has a profound impact on the perioperative period for individuals undergoing cataract surgery [53]. Research conducted in various countries has revealed different HTN prevalence rates: in Greece, patients presenting only with HTN ranged from 43.8% in cases with subcapsular cataracts to 24.3% in cases with nuclear cataracts [47]; in Chandigarh, India, the rate was 4.1% [54]; in Karachi, Pakistan, it reached 43.75% [44]; in Sofia, Bulgaria, the rate was 50% [11]; and in Andhra Pradesh, India, it was reported to be 20.59% [45]. Additionally, a study from Brazil reported a rate of 33% [46]. Concerning the association between HTN and cataractogenesis, Mylona et al. established a correlation between HTN and the severity of cataracts, finding that HTN was present in 43.8% of patients with subcapsular cataracts, 24.3% with nuclear cataracts, 28.6% with cortical cataracts, and 27.6% with mixed-type cataracts [47]. Their results were consistent with those reported in the meta-analysis by Yu et al., which demonstrated that HTN was associated with an increased risk of cataract regardless of cataract type [12]. Moreover, Anitha et al. found that hypertensive patients were 1.7 times more likely to develop cataracts compared to non-hypertensive patients [48]. These results aligned with similar studies conducted on animal models, as shown by Khan et al., who performed an experimental study to explore cataractogenesis mechanisms in hypertensive models. They found that chronic HTN significantly elevates oxidative stress, as evidenced by decreased antioxidant levels and increased serum malondialdehyde (MDA) concentrations. Reduced serum antioxidant levels due to HTN can result in cellular damage to organs, including the eye lens. Their results suggest that increased oxidative stress and ionic imbalance in the lenses are key mechanisms for cataract development, as they have the potential to influence protein content and induce lenticular opacity [49]. Beyond its role in cataractogenesis, HTN also affects the perioperative period, as evidenced by Lira et al., who reported that hypertensive individuals, even those who had a prior history of well-controlled BP, were more likely to experience BP spikes during surgical procedures [51]. Furthermore, elevated BP during ocular surgery may result in various complications, including suprachoroidal hemorrhage, as well as serious systemic issues such as stroke or myocardial ischemic events. Therefore, continuous BP monitoring ensures optimal ocular and systemic outcomes. Proper control of BP before surgery is necessary, and this may involve adjusting antihypertensive therapy to prevent fluctuations that may compromise ocular and systemic health during surgery [52].

Ischemic heart disease (IHD), often called coronary artery disease, is a medical condition characterized by reduced blood flow to the heart muscle tissue. This decrease is usually linked to the constriction or blockage of coronary arteries, primarily caused by pathophysiological changes related to atherosclerosis. This condition causes an imbalance of myocardial oxygen demand and supply, which clinically presents as either angina pectoris or, in a late stage, as myocardial infarction [55]. IHD affects 20–30% of older adult patients undergoing cataract surgery, thus representing an additional risk. In 2001, Hu et al. conducted a prospective study that showed a notable positive correlation between cataract surgery, the incidence of IHD, and overall mortality, with relative risks (RRs) of 1.88 and 1.37, respectively [9]. Subsequently, Hu et al. further evaluated this relationship, finding that patients with cataracts who underwent surgery had a higher observed risk of developing IHD compared with those who did not receive surgical intervention. However, these findings should be interpreted cautiously, as they are likely influenced by shared cardiovascular risk factors and confounding by indication, rather than reflecting a direct causal effect of cataract surgery itself. One possible explanation is that atherosclerosis represents a systemic and progressive process that is not modified by localized ocular surgery but is rather driven by underlying patient-related risk factors [13]. Clinically, patients with existing IHD may experience myocardial stress due to the strain of surgical procedures. This stress can worsen with pre-existing conditions like chronic obstructive pulmonary disease (COPD) and diabetes [56]. Therefore, choosing the correct anesthetic agents is paramount. The simultaneous use of local anesthesia with monitored sedation is preferred over general anesthesia to reduce cardiovascular stress, especially in patients with a history of angina or recent coronary interventions. However, despite the use of local anesthesia, the preoperative assessment should include a thorough evaluation of any recent cardiac symptoms, exercise capacity, and the need for further cardiac evaluation [57,58,59].

Arrhythmias, particularly atrial fibrillation (AF), also complicate the perioperative care of older adults undergoing cataract surgery. Indeed, these patients are at increased risk of perioperative cardiovascular complications, particularly thromboembolic events (e.g., stroke in atrial fibrillation) and arrhythmia exacerbations [60]. AF is detected in nearly 10–15% of patients, especially those undergoing surgery in their seventh or eighth decade of life [61]. In addition, AF is a risk factor for thromboembolic events; thus, anticoagulation therapy—which is typically used for stroke prophylaxis in such patients—carries a risk of bleeding that must be carefully managed during surgery. It is critical to strike a balance between anticoagulation for stroke prevention and the management of the associated risk of bleeding, particularly in patients with valvular heart disease or thromboembolism [62]. Hu et al., in their nationwide cohort study, reported an association between cataract surgery and increased risk of AF. The analysis proved that the subjects who had cataract surgery had a 1.32-fold risk of developing AF, while non-surgical patients with cataracts had a 1.21-fold risk [14]. However, these findings should be interpreted with caution, as they are based on observational data and may reflect shared systemic risk factors and residual confounding rather than a direct causal effect of surgery. Recently, Bikbov et al. conducted a population-based investigation as part of the Ural Eye and Medical Study, revealing that the prevalence of AF was 1.6% [63]. The prevalence rose from 1 in 1029 individuals (0.1%) in the 40-to-49-year age group to 29 in 619 individuals (4.7%) in the 70-to-79-year age group and, finally, to 12 in 159 individuals (7.5%) in the 80-years-or-more age group. A greater prevalence of AF correlated with increasing age, urban residency, positive medical history of CVDs or stroke, a lower incidence of neck pain, elevated serum bilirubin concentrations, a diminished prothrombin index, advanced stages of arterial HTN, and elevated ankle–brachial indices. However, no statistically significant associations were observed between the onset of AF and the degree of nuclear cataract (*p* = 0.50), prevalence of nuclear cataract (*p* = 0.40), degree of cortical cataract (*p* = 0.43), presence of cortical cataract (*p* = 0.17), lens pseudoexfoliation (*p* = 0.58), and status following cataract surgery (*p* = 0.38) [63].

In conclusion, older adults with cardiovascular comorbidities require a comprehensive and multidisciplinary preoperative evaluation. Healthcare providers can improve the safety and outcomes of cataract surgery in this vulnerable population by effectively managing these conditions and tailoring perioperative care to reduce cardiovascular strain [64].

### 2.3. Metabolic Comorbidities

Diabetes and dyslipidemia are common metabolic comorbidities in older adult patients with cataracts, increasing the risk of perioperative and long-term complications [15]. Diabetes, which affects an estimated 20–30% of people over the age of 65 worldwide [65], is one of the most common metabolic disorders in those with cataracts because it promotes cataractogenesis and cataract progression through mechanisms such as oxidative stress and the production of advanced glycation end-products (AGEs) [66]. The onset of cataracts in diabetic patients typically occurs at an earlier stage and progresses rapidly, resulting in a greater need for surgical intervention in this population. Furthermore, inadequate glycemic control increases the risk of intraoperative and postoperative complications, such as delayed wound healing, postoperative infections, and the progression of pre-existing diabetic retinopathy [67]. The pathogenesis of diabetic cataracts primarily results from the activation of the polyol pathway, wherein aldose reductase facilitates the transformation of surplus glucose into sorbitol, which accumulates within the lens due to slow conversion to fructose and its polar characteristics, which impede diffusion out of the lens. Concerning pathogenesis, the “osmotic hypothesis” suggests that osmotic stress from sorbitol accumulation leads to water influx, swelling of lens fibers, and eventual degeneration. Furthermore, it causes apoptosis in lens epithelial cells, further destabilizing lens structure and function. Simultaneously, oxidative stress continues to cause damage to the lens. In addition, the osmotic stress produces reactive oxygen species (ROS), while hyperglycemia increases protein glycation and AGE formation. Therefore, these mechanisms increase the production of ROS, such as hydrogen peroxide and nitric oxide, which contribute to increased cellular damage. Disrupted antioxidant protection, e.g., inactivation of superoxide dismutase (SOD1), predisposes the lens to oxidative injury, thus increasing the opacification of the lens. Cataracts are two to five times more common in diabetic patients than in non-diabetics, and they progress to visually significant stages more quickly. Diabetic individuals usually experience cataract development about ten years earlier than those without diabetes. Numerous studies highlight that those with diabetes not only have a higher prevalence of cataracts but also develop them at a younger age compared to nondiabetic individuals [68,69]. The Wisconsin Epidemiologic Study of Diabetic Retinopathy investigated cataract extraction rates in diabetic patients. After ten years, the cumulative rate of cataract surgery was found to be 8.3% for individuals with type 1 diabetes and 24.9% for those with type 2 diabetes. Key predictors for cataract surgery in type 1 diabetes patients included age, the severity of diabetic retinopathy, and proteinuria. In type 2 diabetes patients, age and insulin use were linked to a higher risk of cataracts [70]. The Beaver Dam Eye Study cohort, comprising 3684 participants aged 43 years and older, revealed a correlation between diabetes mellitus and cataractogenesis, showing that the incidence and progression of cortical and posterior subcapsular cataracts were associated with diabetes. Furthermore, elevated glycated hemoglobin levels were linked to an increased risk of nuclear and cortical cataracts. Thereafter, a complete analysis of the Beaver Dam Eye Study examined the incidence of cataract formation in 4926 adults [71]. Those with diabetes were more likely to have cortical lens opacities and showed evidence of a history of cataract surgery at a higher frequency than nondiabetic patients. Analysis of data confirmed that a longer duration of diabetes was linked to a greater rate of cortical cataracts and a greater rate of cataract surgery [72]. The Blue Mountains Eye Study, a population-based, cross-sectional survey from 1992 to 1994, was designed to examine the relationships among nuclear, cortical, and posterior subcapsular cataracts (PSCs) in a cohort of 3654 participants. The study replicated previous evidence of the adverse effects of diabetes on the lens and found a statistically significant relationship between diabetes and posterior subcapsular cataracts [73]. Beyond perioperative, intraoperative, and postoperative ocular conditions such as dry eye, delayed epithelial healing, myotic pupil size, light reflex disorders, and rapid progression of retinopathy or macular alterations, patients with diabetes face further challenges. These complications are worsened by aging and can be aggravated by harmful interactions with ocular and non-ocular comorbidities. Specifically, older adult diabetic patients with cataracts face a greater risk of systemic infections, including pneumonia, urinary tract infections, wound infections, and bacteremia [74]. In addition, physicians should recognize that anxiety, the type of anesthesia, and surgical trauma significantly contribute to metabolic destabilization, which results in a catabolic drive characterized by enhanced gluconeogenesis and glycogenolysis, subsequently elevating glucose release into the circulation. As a result, physiological stress from surgical procedures, anesthesia, and underlying diseases causes an increase in the secretion of counterregulatory hormones such as cortisol, glucagon, growth hormone, and catecholamines. This physiological response reduces insulin secretion and peripheral glucose utilization, increases insulin resistance, and boosts lipolysis and proteolysis. Consequently, it is critical to ensure optimal diabetes management before, during, and following any surgical procedure to avoid potential complications [75]. In both diabetic and nondiabetic populations, hyperglycemia during the perioperative period is an independent predictor of poor surgical outcomes. Irregular glucose levels during the perioperative phase can jeopardize patient safety and surgical outcomes. Furthermore, protocols for administering preoperative glycemic control vary across institutions, and a universal consensus is still lacking [16]. Several studies regarding the protocol to be followed have been published in the literature [76,77,78,79]. In 2016, Woo et al. [78] surveyed local ophthalmologists and anesthesiologists about their approaches to this prevalent clinical issue, identifying common practice patterns in Singapore. The findings revealed that most clinicians preferred to avoid using oral hypoglycemic agents (82.9%) or insulin (69.8%) and keep the patient fasting before surgery to reduce the risks associated with hypoglycemia. Furthermore, most respondents (85.3%) affirmed the importance of checking blood glucose levels before surgery, while only 34.1% indicated they would monitor blood glucose levels afterward [78]. The Association of British Clinical Diabetologists recommends that patients keep their blood glucose levels within the target range of 6–10 mmol/L, with levels as high as 12 mmol/L. Individuals who use subcutaneous insulin should check their blood sugar three to four times daily when not fasting. Those who only take non-insulin diabetes medications should check their glucose levels at least twice a day. Blood glucose levels should be monitored at least once daily in patients managing their diabetes through diet. Finally, preventive treatment for impending hypoglycemia (for example, in cases where glucose levels are between 4 and 6 mmol/L), hypoglycemia (below 4 mmol/L), and hyperglycemia (more than 13 mmol/L) should be prescribed in advance. A hypobox should be easily accessible from all points of care, including the admission ward, operating room, and recovery area [80].

Besides diabetes, dyslipidemia, obesity, and a high-fat diet have been identified as causes of increased risk for age-related cataracts. Dyslipidemia, which affects nearly 60% of adults aged 65 years and above in the United States, has been recognized as a significant risk factor for cataract development [81,82]. Disrupted lipid metabolism has been studied in close association with the development of cataracts since 1825. Various mechanisms contribute to cataractogenesis, including oxidative stress, lipid peroxidation, and alterations in the structural integrity of lens membranes. In a study conducted in 2018, Li et al. found that patients with age-related cataracts had much higher serum levels of low-density lipoprotein cholesterol (LDL-C), triglycerides (TG), total cholesterol (TC), and apolipoprotein A. Elevated LDL-C (odds ratio (OR) = 1.897) and TG (OR = 1.854) levels were identified as independent risk factors for cataracts in older adults [83]. Additionally, research by Mundra et al. revealed that serum TG and very-low-density lipoprotein (VLDL) levels were significantly higher in cataract patients than in normal controls. In contrast, serum cholesterol, LDL, and high-density lipoprotein (HDL) levels showed no significant differences [84]. Further, Khataminia et al. discovered that cataract patients displayed remarkably elevated levels of TC, TG, and very low-density lipoprotein cholesterol (VLDL-C) compared to the control cohort. In addition, it was found that TC, TG, and VLDL-C levels were further raised in cataract patients diagnosed with pseudoexfoliation syndrome (PEX) compared to those who had cataracts without PEX [85]. Another study conducted by Hiller et al. showed a positive correlation between increased levels of TG and increased risk for PSC in men. This relationship remained significant after adjusting for age and multivariable factors. Moreover, findings demonstrated that men with low HDL-C may face an increased risk of PSC, although this finding reached only borderline statistical significance [86]. Hence, screening and treating dyslipidemias should be a primary step in preventing cataract progression among older adults. These strategies for lifestyle modification should be accompanied by pharmacological treatment as recommended in the 2019 ESC/EAS Guidelines for the Management of Dyslipidemia. Combined with measures to reduce oxidative stress and inflammation, these strategies may further reduce cataract formation [87].

Furthermore, central obesity, HTN, insulin resistance, and dyslipidemia frequently coexist to form a metabolic syndrome (MetS) cluster that increases the risk of age-related cataracts and exacerbates perioperative risks in candidates for cataract surgery. The three main physiological and pathological processes linked to MetS—oxidative stress, osmotic imbalance, and non-enzymatic protein glycation—are responsible for cataractogenesis. To date, MetS has been linked to cataracts in several observational studies [88]. Specifically, according to the Singapore Malay Eye Study, cataracts were more common in patients with MetS than those without it. Their results showed that all three forms of cataracts—nuclear, cortical, and PSC—were associated with high BP. Additionally, diabetes was explicitly linked to cortical and PSCs. In contrast, cortical cataracts were also associated with elevated body mass index (BMI), HDL levels, and MetS. Interestingly, the odds of developing cataracts increased fourfold when diabetes and high BP co-occurred (OR: 4.73) [17]. Research conducted by the Korea National Health and Nutrition Examination Survey revealed that the prevalence of cataract was higher among participants who also had MetS, especially among females. Cataract formation significantly correlated with MetS in females, showing an adjusted odds ratio (aOR) of 1.24. Cataracts in women were also significantly associated with decreased HDL levels, post-fasting hyperglycemia, and hypertriglyceridemia, with adjusted odds ratios (aORs) of 1.27, 1.23, and 1.26, respectively. In addition, MetS and low HDL-cholesterol were associated with nuclear cataract in women, with aORs of 1.25 and 1.25, respectively. No such association was shown among males [89]. Similarly, Galeone et al. and Linblad et al. discovered that MetS, its components, and their combinations were linked to a higher risk of cataract extraction in an Italian hospital population and Swedish women, respectively [18,90]. Therefore, monitoring blood glucose, BP, and lipid levels through appropriate medications is essential in managing MetS and reducing associated complications, including cataracts.

Nowadays, obesity is a significant metabolic risk factor with high prevalence in older adults, which may play an indirect role in cataract surgery outcomes. Specifically, obesity is related to cataracts through various mechanisms, including oxidative stress, inflammation, and endothelial dysfunction, and many inflammatory markers associated with cataracts have been identified, such as C-reactive protein (CRP), intracellular adhesion molecule-1 (ICAM-1), and interleukin-6 (IL-6) [91]. A recent meta-analysis of 16 studies involving a total sample of 1,607,125 showed that higher BMI significantly increased the risk of developing age-related cataracts (RR: 1.18), cortical cataracts (RR: 1.20), and PSCs (RR: 1.44). In addition, according to the dose–response analysis, an increase in BMI by 5 kg/m^2^ was associated with a 6% and 27% increased risk of age-related cataracts (RR: 1.06) and PSC (RR: 1.27), respectively. Moreover, obesity contributes to cataract development through several interrelated mechanisms. Indeed, excessive adipose tissue forms ROS, which lead to oxidative stress, causing damage to lens proteins and finally resulting in opacification. Low-grade chronic inflammation that coexists with obesity adds to this oxidative damage and accelerates lens opacification. Conversely, insulin resistance and elevated blood sugar levels due to obesity lead to additional complications, like the glycation of lens proteins, which further impairs lens transparency. Thus, addressing obesity is essential in lowering the risk of cataracts associated with aging. Managing weight and following a diet rich in antioxidants can help repair oxidative damage to lens proteins. Finally, managing comorbidities linked to obesity, such as diabetes and HTN, is paramount. Indeed, proper regulation of blood glucose and BP can slow the development of cataracts and safeguard overall eye health [92].

### 2.4. Neurological Comorbidities

Neurological comorbidities including Parkinson’s disease, epilepsy and stroke-related complications, such as post-stroke cognitive impairment and motor dysfunction, are common in older adults and significantly impact the perioperative management of age-related cataract surgery in this population. Specifically, these conditions often complicate anesthesia delivery, contribute to postoperative delirium, and reduce compliance with treatment protocols.

Approximately 5% of patients undergoing cataract surgery have a history of stroke [93]. Cataracts and strokes share common risk factors, which increases the likelihood of both conditions occurring together. A retrospective chart review of acute stroke inpatients at Oxford Radcliffe Hospital Trust during 2003–2004 was undertaken to establish a cause-and-effect relationship between stroke and cataract surgery. The study found that the cataract surgery rate among stroke survivors aged 64 years and above was 2.6% compared to the national survey reference [94]. In addition, computed tomography radiation exposure as part of standard management in acute stroke has been proposed to expedite cataractogenesis [19]. However, Huang et al. found that early-onset cataract patients were not at a significantly increased risk of subsequent ischemic stroke development as per the Longitudinal Health Insurance Database 2000, where the researchers found a non-significant 1.48-fold higher risk of ischemic stroke in early-onset cataract patients [95].

Beyond stroke, Parkinson’s disease might be related to cataracts in older adult patients. To explore whether cataracts could be associated with an increased risk of Parkinson’s disease, Lai et al. conducted a retrospective cohort study in 2014 examining the risk of Parkinson’s disease among patients with cataracts. The mitochondrial dysfunction theory suggests there may be an association between cataracts and Parkinson’s disease. Mitochondria are crucial organelles that play a vital role in energy metabolism, calcium regulation, stress response, and apoptosis. Mutations in mitochondrial DNA can disrupt mitochondrial function, leading to reduced energy production, dysfunction of the electron transport chain, and increased production of reactive oxygen species. This can cause oxidative stress, oxidizing cellular components, which may compromise cellular function, damage cells, and lead to apoptosis in both lens and neuronal cells. Thus, mitochondrial dysfunction may partially contribute to the development of cataracts and Parkinson’s disease [96].

Epilepsy, defined by the occurrence of recurrent, unprovoked seizures, and some antiepileptic drugs, such as carbamazepine, phenytoin, and valproate, have been associated with cataractogenesis. In 2010, Hanhart et al. were the first to analyze the prevalence of epilepsy among patients aged over 50 years undergoing cataract surgery, as well as among 25,968 age and gender-matched controls [20]. Significantly, epilepsy had a much higher prevalence in those undergoing cataract surgery, with an odds ratio of 1.4 for men and 1.2 for women. Antiepileptic medications, particularly clonazepam (OR = 1.5) and carbamazepine (OR = 1.4), were prescribed more frequently to patients undergoing cataract surgery. In addition, multivariate logistic regression analysis disclosed a significant correlation between cataract surgery and epilepsy with an odds ratio of 1.26 (*p* < 0.001). Hence, there is a close relationship between the incidence of epilepsy and cataract surgery, which is further strengthened by the use of antiepileptic drugs [20]. Concerning the effects of clonazepam and carbamazepine, these medications directly affect lens channels and the regulatory mechanisms of aqueous humor. Moreover, a relevant mechanism of phenytoin may include folate deficiency, which has protective functions against cortical and nuclear cataracts. In addition, some comorbidities associated with seizures might require therapeutic measures potentially harmful to the lens, such as steroids that may be prescribed to mitigate many systemic diseases and/or the side effects of antiepileptic drugs, such as Stevens–Johnson syndrome and toxic epidermal necrolysis [97,98,99].

Considering the intricate relationship between neurological comorbidities and cataract surgery in older adults, clinicians need to understand the perioperative challenges linked to stroke, Parkinson’s disease, and other conditions such as epilepsy. Thus, an interdisciplinary strategy that includes ophthalmologists, neurologists, and anesthetists is crucial for enhancing perioperative care, reducing complications, and improving these patients’ visual and neurological outcomes.

### 2.5. Respiratory Comorbidities

Respiratory diseases, such as COPD, asthma, and chronic lung disease, are common in older individuals and exert considerable influence on perioperative care in age-related cataract surgery.

Regarding epidemiology, COPD patients have a higher incidence of cataracts than the general population, and cataract patients have a lower quality of life. Additionally, in a study conducted by Irie et al., the incidence of moderate to severe exacerbations over two years was found to be significantly higher in COPD patients with cataracts than in those without cataracts. Their study found that the incidence of cataracts in COPD patients between 70 and 79 years was 53.38%. Surprisingly, they showed that the presence of a cataract was an independent risk factor for subsequent exacerbations, even after controlling for established risk factors for exacerbations. The exact cause-and-effect relationship between cataracts and exacerbations remains unclear. Nevertheless, cataract development and COPD exacerbations may have a shared pathogenesis, possibly driven by oxidative stress [21]. Savran et al., through their meta-analysis, evaluated the extent of association between corticosteroid exposure and cataracts in patients with asthma and COPD and found that daily high-dose inhaled corticosteroid (ICS) carries a significant risk of cataract development. Their meta-analysis specifically showed that, on average, there was a twofold increase in the risk of cataract formation in patients with COPD and asthma who were exposed to corticosteroids. Furthermore, the meta-regression showed a dose–response relationship between the degree of corticosteroid exposure and cataract risk. The authors concluded that high numbers of prescriptions and high daily dosages of corticosteroids considerably increased the risk of cataract development in patients with both asthma and COPD. Specifically, a daily dose of 1000 μg or higher of inhaled corticosteroids significantly increased the risk of cataracts relative to the risk in patients taking a lower daily dose. In addition, patients taking oral corticosteroids had a higher risk of developing cataracts relative to patients taking inhaled corticosteroids alone [100]. Over a decade ago, Weatherall et al. performed a systematic review and meta-analysis investigating the association between corticosteroid exposure and cataract development. The review, focusing on case–control studies evaluating the association between ICS use and risk of cataracts, found that ICS use raised the risk of developing cataracts by approximately 25% for each additional 1000 μg daily dose [100]. In addition, the research by Savran et al. showed a strong dose–response relationship concerning the risk of cataracts related to daily ICS doses of more than 1000 μg. The meta-regression analysis also supported these results, affirming the dose–response relationship between corticosteroid exposure and the risk of cataracts. It was established that a daily dosage threshold of ICS of ≥1000 μg corresponds to a cataract risk comparable to that associated with systemic corticosteroid therapy. Conversely, Wang et al.’s study revealed that the risk of cataract development was almost twice as high in patients treated with systemic corticosteroids compared to patients who received ICS alone [101]. Regarding intraoperative risks in patients with respiratory comorbidities, Ishikawa et al. found that chronic pulmonary diseases, such as COPD, bronchial asthma, postoperative lung cancer, and pulmonary fibrosis, were significantly associated with reduced preoperative corneal endothelial reserve, defined as a cell density of less than 2000 cells/mm^2^ [102]. In 2015, Soler et al. explained that COPD was responsible for reduced endothelial functional reserve and enhanced corneal endothelial vulnerability to intraocular surgical stress [103]. Consequently, patients were likely to experience an increased risk of corneal decompensation during surgeries that compromised endothelial functional reserve, such as cataract surgery [103].

Beyond the potential intraoperative ocular complications, some patients, particularly those with spinal or psychological/cognitive issues, uncontrollable movement/tremor, and severe COPD, can face difficulties in lying flat for extended periods, thus experiencing an unpleasant breathing sensation at rest, further accentuated by orthopnea. Due to positioning issues, surgeons must be aware of the various alternatives to positioning patients who cannot correctly position themselves for cataract surgery, including the “face-to-face” position [23,104]. Finally, cataract surgery also demands a stable surgical field. Uncontrolled movements such as coughing can increase the likelihood of complications, especially among patients with respiratory problems, as coughing could cause increased intraocular pressure and even lead to suprachoroidal hemorrhage [22].

### 2.6. Renal and Hepatic Comorbidities

Renal comorbidities are associated with the onset of cataracts, particularly among older individuals. Specifically, chronic kidney disease (CKD), which is characterized by diminished kidney function or persistent kidney damage lasting three months or longer, shares fundamental pathogenetic mechanisms with various ocular disorders. Microvascular dysfunction, epithelial dysfunction, oxidative stress, and inflammation contribute to this process. In cataractogenesis, advanced glycation end products interact with lens proteins, causing protein crosslinking that leads to lens yellowing and ROS production [105]. Furthermore, patients with CKD may exhibit an elevated risk of cataracts attributable to hypocalcemia, oxidative stress, or increased levels of circulating urea, which leads to water accumulation in the lens. This phenomenon is particularly pronounced in the lens cortex and the posterior subcapsular region, which are most sensitive to these stresses. To date, a multitude of cohort studies have investigated the correlation between cataract formation in patients with CKD and the severity of renal impairment, yielding conflicting results. Klein et al. conducted a five-year prospective study in 1988, concluding that abnormalities in renal function did not demonstrate a significant correlation with the incidence of cataracts when age and other factors were adjusted for gender [106]. In 2005, Huynh et al. conducted a study to examine the relationship between creatinine clearance and the five-year incidence of cataracts and subsequent cataract surgeries [107]. The findings revealed that, when compared to the age-associated incidence of cataract surgery, individuals experiencing moderate or worsening renal impairment at a younger age exhibited higher odds of undergoing cataract surgery. However, these odds were not significantly lower among the older population. The study established a significant correlation between moderate to severe renal impairment and the likelihood of undergoing cataract surgery (OR: 2.75) among participants in their fifties [107]. Recently, Chiu et al. established an age-dependent correlation between heightened renal impairment and the likelihood of undergoing cataract surgery. Their findings indicated that individuals aged under 60 years with moderate to severe renal impairment exhibited increased odds of experiencing cataract surgery, with the probability being significantly greater among younger patients (those below 50 years) in comparison to their older counterparts (50 years and above). This suggested that individuals under 60 years of age with moderate to severe renal impairment are at a markedly elevated risk for requiring cataract surgery [108]. In 2023, Wakasugi et al. analyzed the risk of cataract surgery in non-dialysis and dialysis-dependent CKD patients compared to non-CKD patients [28]. Their results indicated that patients with dialysis-dependent CKD had twice the adjusted risk for incident cataract surgery compared to non-CKD patients. By contrast, non-dialysis-dependent CKD patients had an identical risk profile to non-CKD patients [28]. Recently, Liu et al. conducted a retrospective analysis of data encompassing one million individuals from a health insurance database in Taiwan. Their findings indicated that the risk of cataract development was considerably higher in the CKD group, with an adjusted hazard ratio (aHR) of 1.86 compared to the non-CKD group. The risk was higher for those with end-stage renal disease, as shown by a hazard ratio (HR) of 2.33. This suggested that greater renal impairment correlated with an increased risk of cataract development [109]. In line with the results of Liu et al., Huang et al. explored the relationship between CKD and self-reported cataracts. They stratified the participants by age, categorizing them into those under 60 and those at or above 60 years. Their results showed that, after adjustment for a variety of confounders—such as age, sex, body mass index, smoking status, diastolic BP, and medical histories of HTN, diabetes mellitus, and dyslipidemia—both CKD and serum albumin were significantly related to the development of cataracts [110]. Beyond the established association between CKD and age-related cataract onset, a few studies evaluated the intra- and perioperative risks of cataract surgery in CKD patients. Indeed, patients with end-stage renal disease (ESRD) were found to be more likely to experience problems such as bleeding, infection, and perioperative mortality after undergoing major surgery [29,111]. In 2020, Hsiao et al. analyzed the risk of cataract surgery-related complications in patients receiving dialysis and compared this risk to that in patients with CKD or ESRD [111]. They found that patients with ESRD had a significantly higher risk of vitreous hemorrhage (VH) and reoperation for dropped nucleus or vitreous complications after cataract surgery than patients without ESRD after adjusting for the use of antiplatelet drugs/anticoagulants and hospital level. The increased risk of VH and reoperation for dropped nucleus or vitreous issues after cataract surgery in ESRD patients might be related to structural alterations in the eye, dynamic eye changes during hemodialysis, or a predisposition to bleeding. An additional outcome of heightened corneal edema following cataract surgery in nondiabetic ESRD patients may be attributed to structural abnormalities in corneal endothelial cells, which show greater sensitivity to injury [111].

Liver diseases, such as non-alcoholic fatty liver disease (NAFLD), alcoholic liver disease (ALD), and liver fibrosis and cirrhosis, which involve metabolic changes and inflammation, can elevate the risk of cataract development because of these metabolic and immune system alterations [112]. The first systematic study to examine the potential correlation between severe liver diseases, such as NAFLD, ALD, viral hepatitis, liver fibrosis, and cirrhosis, and an elevated risk of cataract development was conducted in 2024 by Chen et al. [113]. They included 326,558 participants using the UK Biobank, a sizable prospective cohort study that recruited over 500,000 UK residents between 2006 and 2010, between the ages of 37 and 73. They found 37,064 people with cataract development after a median follow-up of 13.3 years. When exposure was treated as a binary time-varying variable, people with severe NAFLD (HR: 1.47), ALD (HR: 1.57), and liver fibrosis and cirrhosis (HR: 1.58) had a higher risk of cataracts, but not people with viral hepatitis (*p* = 0.13) [113]. In 2017, Park et al. demonstrated a strong association between hepatitis virus infections, liver damage, and cataract formation, revealing that the ORs for nuclear and overall cataracts linked to hepatitis B virus (HBV) infection were 1.09 and 1.07, respectively, compared to those without HBV infection; for hepatitis C virus (HCV) infection, the ORs were 1.35 and 1.40, respectively [30]. While the exact biological mechanisms remain unknown, changes in the lens microenvironment resulting from metabolic shifts linked to NAFLD and ALD could play a role in cataract development [114]. Both ALD and NAFLD commonly show metabolic changes. For example, in human lens epithelial cells, these conditions can increase circulating cysteine levels, potentially causing endoplasmic reticulum stress and facilitating the production of ROS. In NAFLD and ALD, reduced glutathione levels may lead to crystallin degeneration and lens opacification. Furthermore, a deficiency in vitamin D, which is also observed in patients with these two liver diseases, may contribute to cataractogenesis [115]. In addition to metabolic factors, systemic inflammation, indicated by elevated proinflammatory cytokines, may result from the immune response activation in both NAFLD and ALD [116]. Furthermore, immune cells that have exited the vasculature and migrated to the lens may also play a role in the development of cataracts, as recently evidenced in animal models. Since liver disease is characterized by elevated levels of proinflammatory cytokines, which increase vascular permeability, facilitate transendothelial migration, and enhance leukocyte-endothelial adhesion, it may indirectly help immune cells infiltrate the lens and accelerate cataract formation [117]. The connection between liver fibrosis, cirrhosis, and cataracts has been evidenced by elevated serum ceramide levels in animal models, potentially leading to ROS production and apoptosis in human lens epithelial cells [118]. Alterations in Hcy and methionine metabolism might contribute to the formation of cataracts. Conditions such as liver cirrhosis and fibrosis, stemming from various liver diseases, create a more intense inflammatory environment [119].

Given the intricate relationship between renal and liver comorbidities and cataract surgery, healthcare providers need to be aware of the perioperative difficulties linked to CKD, ESRD, and liver conditions like NAFLD and ALD. A collaborative strategy that includes ophthalmologists, nephrologists, hepatologists, and anesthetists is crucial for refining surgical planning, reducing complications, and improving visual and overall health outcomes in these high-risk patients.

### 2.7. Psychiatric Disorders

Mental health is essential to ensure overall well-being, especially for older adults [24]. According to currently available research, concomitant psychiatric disorders and cataracts in older adults lead to decreased physical activity, decreased social engagement, and a higher risk of psychiatric conditions, such as anxiety and depression. Consequently, vision impairment due to cataracts could worsen such conditions, triggering deterioration in autonomy, increased social isolation, and poorer general mental health outcomes. Additionally, the relationship between cataract surgery and psychiatric disorders is bidirectional, with preoperative mental health playing an essential role in determining the success of the operation. Therefore, it is paramount to explore and critically evaluate the intricate relationship between cataracts and psychiatric disorders to develop comprehensive care plans tailored to the needs of older adults [120].

Concerning the epidemiology and risk factors associated with psychiatric disorders in older adults with cataracts, several studies have explored the role of anxiety or depression [25,121,122]. In their 3-year prospective cohort study conducted in 2021 using data from the Canadian Longitudinal Study on Aging, Grant et al. discovered that cataracts were linked to incident depressive symptoms (RR = 1.20) [121]. Eramodugolla et al. reported that anxiety was significantly related to self-reported cataracts, reduced low-contrast visual acuity, motion sensitivity, and contrast sensitivity [122]. In 2024, Wang et al. examined the relationship between cataract surgery and mental health in older adults, discovering a strong correlation between increased symptoms of anxiety and depression and vision impairment [25]. Many factors contribute to anxiety and depression in patients with cataracts. Significant concerns include the risk of blindness, financial burdens, a deterioration in quality of life due to limited physical activity, barriers to effective communication, and a lack of education that may hamper understanding of medical terminology [25]. Chen et al. found that cataract patients had a significantly higher risk of depression (HR = 1.72) compared to the non-cataract control group, even after adjusting for potential confounding variables. Furthermore, compared to individuals without cataracts, the HR increased to 2.14 for cataract patients who did not receive surgical intervention. In their comprehensive 16-year nationwide cohort study, they found that cataract surgery reduced the risk of depression by 25% when compared to non-surgery groups [123]. Different studies have been performed to analyze the strength of association between depression and cataracts in older adult patients worldwide [124,125,126,127]. In a community-based survey of 4611 Chinese adults aged 60 or older, Wang et al. (2016) performed the first large-scale population-based study to evaluate the relationship between age-related cataracts and the presence of depressive symptoms [127]. They observed that adults with cataracts were more likely to experience depressive symptoms than those without (OR = 1.33) [127]. In their multicenter prospective longitudinal cohort study, Kumar et al. discovered that 56.6% of adults awaiting cataract surgery had comorbid depressive and anxiety symptoms. Notably, those who reported neglect or mistreatment by family and friends had significantly higher mean depression scores. Individuals who reported being neglected or mistreated by family and friends had the highest mean scores for general anxiety. The subsequent demographic category in terms of prevalence consisted of individuals aged 70 and above and those visually impaired [126]. Their findings were consistent with previous research, which found that low educational attainment, gender, diminished quality of life, the presence of comorbid conditions, and poor vision acuity are major contributors to depression and/or anxiety in patients with untreated cataracts [124,125].

Given the high incidence of psychiatric disorders in older adults, early cataract surgery can optimize mental health outcomes through the restoration of vision, confidence building, activity enhancement, and lessening of social isolation and risk of injury. The most substantial evidence for the impact of cataract surgery on depression comes from a randomized controlled trial in the United Kingdom. Although the study revealed a slight improvement in depressive symptoms after first-eye surgery, it was restricted to women aged over 70 [128]. A later small prospective study conducted in the United Kingdom, involving 46 patients who underwent cataract surgery on their first eye, revealed a considerable decrease in depressive symptoms after either the first surgery, the second surgery, or both procedures [129]. Compared to this, a small Australian randomized controlled trial involving 25 cataract surgery patients and 20 controls revealed no significant reduction in depressive symptoms after the first-eye cataract surgery [26]. A longitudinal study in the United States investigated 122 cataract patients who underwent surgical intervention compared to 92 patients who did not. The study’s findings indicated no significant difference in the prevalence of depressive symptoms between these two groups [36]. Meuleners et al. observed in 2013 that there was a notable 18.80% reduction in mental health contacts for anxiety and/or depression in the year following cataract surgery when compared to the year before surgery [130]. Mylona and colleagues, in a recent study involving 150 consecutive patients undergoing phacoemulsification surgery, recorded a statistically significant improvement in depressive status before and after surgery. It was observed that apprehensions about the future, including skepticism regarding potential outcomes and challenges in decision-making, diminished markedly after surgery [27]. Therefore, thorough and meticulous management of depression and anxiety is essential to maximize cataract surgery outcomes in older adult patients, regardless of the immediate effect of the surgery on these disorders. Preoperative education can be crucial for patients and caregivers to fully understand cataracts and their treatment options. Indeed, such knowledge makes it easier to minimize uncertainties and enhance confidence, which are both vital for reducing fear and anxiety related to surgery. Thus, a multidisciplinary team of ophthalmologists, nurses, psychologists, social workers, educators, and occupational therapists can help strengthen preoperative communication strategies with patients [131,132].

## 3. Epidemiology of Geriatric Syndromes

Geriatric syndromes, encompassing frailty, cognitive impairment, sensory deficits, and functional decline, constitute a complex and interrelated array of challenges that can substantially impact the perioperative period and the outcomes of cataract surgery in older adults.

### 3.1. Frailty and Functional Decline

Frailty and functional decline have significant effects on the surgical outcomes and recovery of older adults undergoing cataract surgery. Notably, frailty has been defined as a state of increased vulnerability that occurs due to decreased physiological reserves, and it is found in approximately 10–25% of older adults and up to 50% of individuals aged over 80 years [133]. It is characterized by adverse health outcomes, such as extended hospital stays, increased postoperative complications, and mortality [134]. Frail patients exhibit inferior surgical outcomes compared to non-frail patients due to their diminished capacity to manage perioperative stressors. Frail patients experience elevated odds of postoperative complications, including infections, disturbances in wound healing, and a heightened vulnerability to postoperative delirium. Moreover, functional decline signifies a reduction in physical capacity, adversely affecting an individual’s ability to perform daily activities [135]. The relationship between frailty and cataracts has been extensively studied. In 2006, Klein et al. discovered that every type of lens opacity was associated with at least one frailty indicator in both genders, even after controlling for age [136]. Subsequently, Chen et al. demonstrated that, in the context of age-related cataracts, a significant difference exists only between the non-frail and prefrail cohorts among Taiwanese adults 65 and older. They discovered that cataracts could lead to frailty and emphasized the importance of early diagnosis and treatment of cataracts to avoid the onset of frailty [137]. In 2013, Pathai et al. reported a significant correlation between the severity of frailty and the severity of cataract opacity [31]. They found this relationship by examining biological changes in lens and skeletal muscle protein structures indicative of the aging process in the human body; as a result, age-related cataracts and frailty may share a risk factor [31].

Ghanbarnia et al. recently conducted a cross-sectional population-based study with 1136 participants aged 60 and older. They were the first to discover that cataracts were significantly associated with cognitive frailty (OR 1.66), which is defined as the presence of both cognitive impairment and physical frailty, excluding concurrent dementia [138]. Therefore, in line with previous studies, cataracts may be regarded as an indicator of overall decline in function in older adults. As a result, adults with cataracts may benefit from general function evaluation and assistance. Furthermore, given the implications of frailty and functional decline, a multidisciplinary approach is essential in the preoperative evaluation of candidates for cataract surgery. A better understanding of a patient’s general state can be achieved when geriatric evaluation and functional assessment are integrated into anesthesia and ophthalmology. Older adults would benefit from safer surgical procedures and achieve better recovery outcomes due to such interdisciplinary collaboration, which can result in targeted and tailored interventions such as physical therapy and nutritional support that improve resilience and functional ability before surgery [133,138].

Notably, while frailty and comorbidities increase perioperative vulnerability, it is equally relevant to consider the potential benefits of timely cataract surgery in older adults. Beyond improvements in visual acuity, cataract surgery has been associated with meaningful gains in functional status and patient-centered outcomes. A recent systematic review and meta-analysis demonstrated that first-eye cataract surgery significantly reduces the frequency of falls in older adults with bilateral cataracts (relative risk reduction up to 34%), highlighting its role in preventing injury and maintaining independence [139]. In parallel, emerging evidence suggests that cataracts are associated with an increased risk of fractures, whereas pseudophakia is associated with a lower fracture risk compared with phakic individuals, supporting a protective effect of surgical intervention on injury-related outcomes [140]. Consistent with these findings, earlier studies have demonstrated that cataract surgery significantly improves vision-related quality of life, including better visual function, increased confidence in daily tasks, and enhanced social and emotional health [141].

### 3.2. Cognitive Impairment

Proper vision is critical for the physical and intellectual abilities of all individuals [142]. Cognitive impairment is a prevalent condition among older adults, characterized by cognitive function challenges, which may include memory loss, difficulties with concentration and task completion, problem-solving challenges, comprehension issues, recall struggles, and the incapacity to perform specific commands often associated with alterations in mood or behavior, diminished motivation, and increased sensations of disorientation [143]. Currently, cognitive test scores and visual acuity are closely related, as indicated by longitudinal studies that show that changes in vision and cognition are strongly correlated [144]. Moreover, visual impairment is strictly associated with cognitive decline, as shown by three potential hypotheses that aimed to explain this correlation. The cognitive resource theory presumes that visual impairment raises the cognitive effort required to process visual stimuli, consequently lowering the resources for tasks such as memory and problem-solving. The common factor theory suggests that cognitive and visual decline share a common cause, possibly related to aging or neurodegenerative processes. Lastly, the combined mechanism hypothesis acknowledges the co-occurrence of the two conditions but suggests that both mechanisms may play a role in their co-association. These findings emphasize the need to measure and correct visual impairment before cognitive testing to ensure proper assessments [143]. Despite the strong association, it is challenging to quantify the number of older adults suffering from both cataracts and related cognitive impairment. Nonetheless, several meta-analyses have shown a strong association between them [32,33,145]. In 2023, Xiong et al. examined the relationship between cataracts and cognitive impairment by conducting a systematic review and meta-analysis of observational studies, emphasizing older adults’ risk of cognitive decline [114]. In their meta-analysis of 798,694 participants, they found that the risk of all-cause dementia was greater in older adults with cataracts (pooled HR: 1.22) than in those without cataracts [114]. In addition, cataracts were also linked to an increased risk of Alzheimer’s disease (pooled HR: 1.18). Additionally, cataract patients had a greater risk of vascular dementia (pooled HR: 1.21). An increased risk of mild cognitive impairment in cataract patients was also observed (pooled HR: 1.30) [114]. One year later, Wang et al. performed a systematic review and meta-analysis of articles published between 2010 and 2022 involving 489,211 participants from 10 countries. Their results established a strong correlation between cataracts and cognitive impairment, with a combined OR of 1.32 [34].

Given this significant connection, Yeo et al. conducted a systematic review and meta-analysis to explore whether cataract surgery could reduce the risk of cognitive impairment. In their systematic review and meta-analysis of 24 studies involving 558,276 participants, they found that patients who received cataract surgery had a 25% decrease in the risk of long-term cognitive impairment and dementia compared to those with uncorrected cataracts [146]. Indeed, a reduction in visual input caused by cataracts can lead to structural decay of the brain [147]. Hanson et al. explained that participants with visual impairment, subjected to a longer follow-up duration, demonstrated considerable cortical atrophy defined by a decline in cortical thickness across the occipital lobe. Thus, ocular diseases, such as cataracts, might induce atrophic degenerative processes within the visual cortex, accelerating neuropathological changes in the brain. They also demonstrated further that treatment of participants with antiangiogenic therapy led to stabilization of visual acuity and cortical thickness. Cataract-related visual impairment diminishes cognitive reserve among patients, possibly increasing their risk for dementia and cognitive decline [148]. From the perspective of Yeo et al., two conclusions could be drawn. Firstly, subjects who underwent cataract surgery exhibited risks for dementia and cognitive impairment equal to those of healthy participants with no cataracts. This implies that cataract surgery may eliminate the excess risk of cognitive decline from cataract status. Second, cataract surgery was not associated with changes in short-term cognitive test scores among those with pre-existing cognitive impairment. This suggests that the cognitive benefits of cataract surgery are most relevant to the prevention of dementia, and patients derive the most significant benefit from early treatment before the onset of cognitive impairment [146]. Thus, considering the large number of older adult patients attending ophthalmology services, it is likely that several patients with cognitive impairment have visual symptoms. Therefore, promptly identifying cognitive impairment before surgery could allow clinicians to apply tailored measures to reduce related risks, such as involving family members or caregivers during the preoperative and postoperative periods, which can significantly improve compliance with medical advice and enhance recovery monitoring. In some cases, the surgical staff may modify the perioperative care plan to alleviate perceived stressors, optimizing anesthesia policies or reducing sensory overload in the recovery area. Finally, interprofessional collaboration among multidisciplinary teams, primarily geriatricians and anesthesiologists, is critical for managing the needs of cognitively challenged patients requiring cataract surgery, improving patient safety and optimizing outcomes.

## 4. Public Health Implications and Recommendations

Due to the increase in global life expectancy, the prevalence of age-related cataracts is concurrently rising, presenting significant global challenges to healthcare systems. Despite advances in surgery, various systemic comorbidities affecting older adults present substantial challenges to management, safety, and outcomes. The burden of conditions such as CVDs, diabetes, frailty, cognitive impairment, respiratory disease, renal impairment, and mental illness necessitates a global, multidisciplinary surgical solution. Hence, a public health strategy incorporating focused interventions, enhanced preoperative screening processes, fair policy reforms, and research programs could improve patient outcomes, reduce healthcare inefficiencies, and avoid unnecessary utilization of resources.

### 4.1. Preoperative Screening and Multidisciplinary Management

While cataract surgery is generally safe, older adult patients often face risks due to comorbidities, frailty, cognitive changes, and polypharmacy, which can increase the likelihood of complications and adverse outcomes. Recent reviews have clarified that preoperative assessment for cataract and ophthalmic surgery should go beyond routine medical clearance to incorporate both ophthalmic and systemic factors. Specifically, See et al. pointed out that modern cataract preoperative evaluation should include visual acuity, functional impairment, ocular comorbidities, risk stratification, and proper surgical counseling [149]. Likewise, Macias et al. reported that perioperative assessment in eye surgery should address systemic comorbidities, medication management, anesthesia planning, and factors influencing patient cooperation, positioning, and safety during surgery [150].

Despite the need to investigate and assess the multiple comorbidities that can interfere with the outcomes of cataract surgery in older adult patients, since the early 2000s, substantial evidence has shown that performing routine preoperative testing for every patient does not improve surgical outcomes. This is supported by Keay et al., who included more than 21,000 cataract surgeries in their meta-analysis and found no significant difference in intraoperative or postoperative medical adverse events between patients undergoing routine preoperative testing and those receiving selective or no testing (odds ratio 1.00, 95% confidence interval 0.86–1.16) [151]. Nevertheless, older adult patients still frequently undergo redundant medical assessments, often driven by institutional practices rather than patient-specific clinical indications.

In 2024, Rung et al. investigated the rate and cost of tests for low-risk cataract surgery in patients 65 years and older, aiming to determine predictors of preoperative care and its impact on postoperative complications [152]. They discovered that the surgical facility and members’ care teams were significant determinants of the likelihood of receiving preoperative care instead of patient characteristics or medical indications. The study found no protective effect of preoperative testing against adverse outcomes [152]. Their findings aligned with Chen et al.’s findings, which showed that preoperative testing before cataract surgery was prevalent and more directly related to provider practice habits than patient characteristics [153]. Therefore, delivering care focused and tailored to patients while adjusting for risks to enhance surgical results and ensure safety may represent the future in managing older adults undergoing cataract surgery. In 2021, Cuttitta et al. aimed to ascertain the viability of an individualized preoperative medical screening protocol for cataract surgery [154]. An interdisciplinary group of surgeons, anesthesiologists, physician assistants, and triage nurses developed a risk assessment questionnaire. Low-risk patients did not need an in-office physical examination by the surgeon. By contrast, the moderate- and high-risk patients received a physical examination by a surgeon on the day of surgery, consisting of vital signs and cardiopulmonary assessments. All patients, independent of their risk, also received at least two physical examinations from anesthesiology staff on the day of surgery. The results showed no intraoperative complication rates, 7-day inpatient admission rates, or same-day cancelation rates that substantially differed between control and intervention groups. However, there were more case delays in the control group. Their findings indicated that a risk-based strategy in assessing patients for preoperative cataract surgery led to safe and effective outcomes. No rise in intraoperative complications or inpatient admissions was observed during the post-intervention period, highlighting the value of personalizing preoperative evaluations to eliminate unnecessary tests while ensuring patient safety [154]. Their results aligned with those of Benoit et al., indicating that low-risk patients screened with a preoperative questionnaire did not experience an increase in adverse medical events when a preoperative history and physical examination were omitted [155].

Nonetheless, while avoiding unnecessary assessment is essential, more organized and multidisciplinary perioperative care and a more stringent preoperative system are critical to address systemic health, functional status, and cognitive status evaluations. In 2024, Cao et al. constructed a scientific preoperative assessment program for cataract day surgery for geriatric patients, emphasizing screening for frailty, assessment of cognitive impairment, and medication reconciliation [156]. Using a Delphi method, a consensus evaluation system was created in three broad areas: systemic health assessment, age-related risk factors, and appropriateness for ophthalmic surgery [156]. By methodically assessing essential parameters, such as functional capacity, cognitive status, anticoagulant therapy, and ocular comorbidities, their strategy eliminated unnecessary preoperative testing, decreased day-of-surgery cancelations, and increased perioperative efficiency without jeopardizing patient safety. Furthermore, their results validated the adoption of a multidisciplinary team consisting of ophthalmologists, anesthesiologists, geriatricians, internists, and perioperative nursing personnel to evaluate surgical risk and optimize preoperative care in an integrated manner. This strategy is particularly significant from a public health standpoint, as it enables targeted interventions to reduce perioperative complications, hospital readmissions, and long-term disabilities in older adult patients. From a public health perspective, maximizing preoperative screening and multidisciplinary collaboration yields a twofold benefit: enhancing patient outcomes and reducing system inefficiencies. Implementing risk-based models for assessment promises to reduce some strain on health systems by eliminating unwarranted consultations, refining surgical procedures, and improving the use of resources. In addition, integrating frailty and cognitive screening into standard preoperative assessments can dramatically improve postoperative recovery, decrease delirium and fall complications, and enhance the quality of life among older populations. For example, a frail older adult with stable coronary artery disease and mild cognitive impairment may benefit from a geriatric assessment to optimize nutrition and functional support, as well as cardiology input to manage antiplatelet therapy perioperatively. Meanwhile, coordination with anesthesiology ensures a tailored anesthesia plan to minimize the risk of delirium. Another example involves an older adult with COPD, hearing impairment, and mild frailty, who may require a preoperative pulmonary function assessment and optimization of inhaled therapies, audiological support to ensure effective communication during perioperative counseling, and a tailored anesthetic plan to minimize respiratory depression. Postoperatively, early mobilization and respiratory physiotherapy would be essential to reduce the risk of pulmonary complications and facilitate recovery.

Future research endeavors must improve predictive risk stratification tools, test the cost-effectiveness of multidisciplinary screening models, and examine the broader impact of optimized preoperative pathways on healthcare sustainability and patient-centered outcomes.

### 4.2. Addressing Disparities in Surgical Access and Outcomes

Despite current research highlighting the need for customized preoperative evaluations and multidisciplinary collaboration, disparities in access to cataract services still impact the management of older adult patients. These disparities, particularly among older adult patients in low-income countries and rural areas with multiple comorbidities, represent a serious public health issue. Many older adult patients often face socioeconomic and geographic barriers that lead to delays in treatment, increased visual impairment, and decreased quality of life.

In this regard, the Effective Cataract Surgical Coverage (eCSC) concept, defined as the proportion of those who had undergone a successful cataract surgery relative to all those who either received or required surgery, has revealed several inequities worldwide. Low-income countries continue to struggle with access to surgical care, with women experiencing lower surgical coverage rates in every region than men [157]. Many studies highlighted these disparities worldwide. In 2016, Mundy et al. emphasized how income, education, and health infrastructure affect cataract prevalence and management [158]. Furthermore, patients with low incomes often seek care at more advanced stages of the disease due to delayed access to necessary medical services [158]. Likewise, Moustafa et al. showed that patients from lower socioeconomic backgrounds in the United States were much more likely to be absent from postoperative follow-up visits, resulting in increased risks of undiagnosed complications and poor visual outcomes [159]. Their findings were consistent with those reported by McCormick et al., who found that rural populations also experienced several “barriers,” such as long distances to travel, absence of specialist ophthalmic services, and financial limitations, further restricting access to cataract surgery.

To bridge these gaps, public health initiatives must focus on equitable access through community-based outreach programs, teleophthalmology, and new technologies and innovations [157].

Incorporating teleophthalmology for cataract assessments in older adult patients provides significant potential to improve access to treatment, especially for those facing mobility challenges or residing in underserved regions. However, teleophthalmology is utilized more frequently to assess retinal diseases and less frequently for cataract evaluation due to the current practice of using a torchlight in screening camps following a brief training that does not require highly specialized staff [160]. Notwithstanding, despite being moderate, its use for cataract evaluation has been documented. Recently, Zahlmann et al. reported that teleconsultation in the perioperative management of cataracts reduced the necessity for face-to-face visits and enhanced patient satisfaction and trust regarding their treatment [161]. Likewise, in 2020, Fatehi et al. highlighted that teleophthalmology improved access to specialized ophthalmic services, reducing unnecessary in-person visits and relieving the burden on healthcare facilities [162]. Despite the several challenges in optimizing service use for the older adult population, incorporating teleophthalmology into cataract care for older adults can enhance access to timely assessment and management, especially among those living in underserved communities or with mobility constraints [162].

Regarding innovations for management of age-related cataract surgery in underserved areas, in 2017, a comprehensive study conducted by Imtiaz et al. among remote Indian villagers assessed the efficacy of the Sankara Electronic Remote Vision Information System (SERVIS), an Android-based tablet application that captures patient ocular health data, along with systemic comorbidities and socio-demographic information, including age, gender, education, occupation, and family income. They noted significant benefits compared to traditional in-person screening methods, as SERVIS achieved a much higher conversion rate of referred patients with eye disorders to the campsite. This success was accompanied by reduced screening costs, attributed to SERVIS’s increased referral rate and improved patient turnout at the camp site [163]. Nowadays, artificial intelligence (AI) and machine learning advances are revolutionizing cataract screening and surgical planning [164]. In this context, AI offers a revolutionary opportunity for cataract treatment, especially in older adult populations with poor access to ophthalmic care. Indeed, AI-based cataract screening initiatives have proven to be highly accurate in diagnosis, with convolutional neural networks enhancing cataract detection and grading [165]. These advancements may lead to widespread screening initiatives, reducing disparities in surgical access and facilitating early intervention. In addition, computer-aided surgical planning and postoperative care software, such as smartphone-enabled follow-up apps like CC-Guardian, offer remote patient management with reduced in-person clinic visits and improved long-term results [166]. Despite these advances, evidence from real-world studies, including validation of the CC-Cruiser artificial intelligence system in China, indicates difficulty in transferring effectiveness from the laboratory to the clinic and calls for continued refinement and optimization in various healthcare settings [167].

Beyond innovations, outreach programs (i.e., the coordinated initiatives designed to provide healthcare services to underserved populations) have enhanced uptake of surgical care in resource-poor areas by making services more accessible to high-priority populations. For instance, India’s “Gift of Vision” program at Sankara Eye Hospital uses a doorstep-to-doorstep model of outreach eye care, delivering excellent eye care services to rural areas. Through the implementation of screening camps, the “Gift of Vision” initiative aims to identify patients who have not sought medical attention at hospitals or clinics due to the lack of accessible local eye care facilities, economic constraints, or, in most instances, a lack of awareness regarding eye care. In the screening camps, while patients primarily undergo screening for ocular diseases and cataract surgery, they also receive an initial checkup for BP and glycemia. This ensures that those with HTN or impaired blood glucose are aware of their comorbidities and can seek further care [168,169].

### 4.3. Future Directions for Epidemiologic Research

Epidemiological and public health research can contribute profoundly to planning public health action for age-related cataracts to enhance surgical safety, cost-effectiveness, and long-term health outcomes. Recent data show that cataract surgery improves vision and offers broader benefits, including neuroprotective effects that reduce the risk of cognitive decline. In addition, recent meta-analyses have shown that cataract surgery is linked to a 25% lower risk of long-term cognitive decline, suggesting that vision impairment due to cataract is a modifiable risk factor for cognitive decline [147]. In 2024, Dinarvand et al. emphasized that visual impairment exacerbates physical and mental decline, increasing the likelihood of falls, hospitalization, and dependence on healthcare services [170]. Hence, prospective public health investigations should focus on incorporating vision care as part of geriatric healthcare strategies, given the close connection between visual loss, frailty, and dementia in older individuals. This requires collaboration among ophthalmologists, geriatricians, and public health policymakers to develop strategies focused on early cataract detection and timely surgical intervention [164,165]. In addition, considering the impact of AI in cataract screening and surgical planning regardless of the clinical setting (rural vs. urban), future research in public health will need to examine the cost-effectiveness of AI-supported teleophthalmology, algorithmic bias, and the formulation of policies for fair implementation. Indeed, implementing AI into national eye health initiatives can enhance surgery efficiency, decrease healthcare costs, and improve vision-related quality of life among aging populations globally.

Ultimately, conducting economic analyses of cataract surgery is essential to evaluate the long-term cost benefits of early intervention, particularly in reducing fall-related accidents, fractures, and hospitalizations among older adults. Given that visual impairment constitutes a significant risk factor for falls and the subsequent loss of independence, understanding the broader economic impact of untreated cataracts on healthcare expenditures can guide policy decisions and justify investment in cataract surgical programs as an effective public health intervention. As global populations continue to age, combining epidemiologic research and clinical practice will be required for future public health policy direction, optimizing cataract surgical outcomes, and ensuring access to vision care for all individuals.

The primary strength of our research lies in its thorough epidemiological examination of systemic comorbidities and geriatric syndromes among older adults undergoing cataract surgery, merging clinical insights with public health perspectives. Indeed, translating epidemiological knowledge into practical guidance can help clinicians manage older adults with cataracts, supporting safer and more effective surgical care in real-world clinical settings. Nonetheless, this study has several limitations. First, as a narrative review, it lacks a systematic review’s methodological rigor and reproducibility. Second, article selection was based on non-systematic database searches, which may have introduced selection bias or led to the omission of relevant literature. Third, the available evidence predominantly reflects data from high-income or middle-income countries, which may not be fully generalizable to low-resource settings where cataract burden and access to care differ significantly. Finally, although the review encompasses a wide range of comorbidities, certain conditions, such as malignancies or other systemic diseases, were not covered in depth due to scope constraints.

## 5. Conclusions

Addressing the public health challenge of age-related cataracts requires a thorough epidemiological understanding of related risk factors and comorbidities. It demands a multidisciplinary approach and a well-coordinated public health strategy emphasizing early detection, comprehensive perioperative care, and personalized interventions to enhance patient safety and surgical outcomes. Furthermore, reducing access gaps, enhancing surgical safety, and leveraging AI innovations can significantly improve patient outcomes while lessening the long-term effects of visual impairment. Therefore, fostering interdisciplinary collaboration and implementing evidence-based interventions are crucial for advancing ocular and systemic health among older adults with cataracts worldwide.

## Data Availability

No new data were created or analyzed in this study.
